# Characterization of the Belowground Microbial Community in a Poplar-Phytoremediation Strategy of a Multi-Contaminated Soil

**DOI:** 10.3389/fmicb.2020.02073

**Published:** 2020-08-25

**Authors:** Anna Barra Caracciolo, Paola Grenni, Gian Luigi Garbini, Ludovica Rolando, Claudia Campanale, Giorgia Aimola, Manuel Fernandez-Lopez, Antonio José Fernandez-Gonzalez, Pablo José Villadas, Valeria Ancona

**Affiliations:** ^1^National Research Council, Water Research Institute, Montelibretti (Rome), Italy; ^2^Department of Ecological and Biological Sciences, University of Tuscia, Viterbo, Italy; ^3^National Research Council, Water Research Institute, Bari, Italy; ^4^Consejo Superior de Investigaciones Científicas (CSIC), Zaidin Experimental Station, Granada, Spain

**Keywords:** bioremediation, polychlorobiphenyls, heavy metals, nature based solution, DNA and cDNA Illumina sequencing

## Abstract

Due to their widespread use in industrial applications in recent decades, Polychlorobiphenyls (PCBs) and heavy metals (HMs) are the most common soil contaminants worldwide, posing a risk for both ecosystems and human health. In this study, a poplar-assisted bioremediation strategy has been applied for more than 4 years to a historically contaminated area (PCBs and HMs) in Southern Italy using the Monviso poplar clone. This clone was effective in promoting a decrease in all contaminants and an increase in soil quality in terms of organic carbon and microbial abundance. Moreover, a significant shift in the structure and predicted function of the belowground microbial community was also observed when analyzing both DNA and cDNA sequencing data. In fact, an increase in bacterial genera belonging to *Proteobacteria* able to degrade PCBs and resist HMs was observed. Moreover, the functional profiling of the microbial community predicted by PICRUSt2 made it possible to identify several genes associated with PCB transformation (e.g., *bphAa*, *bphAb*, *bphB*, *bphC*), response to HM oxidative stress (e.g., catalase, superoxide reductase, peroxidase) and HM uptake and expulsion (e.g., ABC transporters). This work demonstrated the effectiveness of the poplar clone Monviso in stimulating the natural belowground microbial community to remove contaminants and improve the overall soil quality. It is a practical example of a nature based solution involving synergic interactions between plants and the belowground microbial community.

## Introduction

Polychlorinated biphenyls (PCBs) are highly stable, hydrophobic and persistent organic pollutants, which have been used for a wide range of industrial purposes, but also as pesticide additives. Due to their recalcitrance to degradation and their lipophilic nature, they bioaccumulate and biomagnify through the food chain and have a broad range of toxic effects on humans, including as probable carcinogens and endocrine disruptors (Turrio-Baldassarri et al., 2007; Letcher et al., 2010; Quinete et al., 2014). Although their production has been banned since the 1970s, their extensive use and high persistence have caused widespread environmental contamination. Consequently, their removal from contaminated ecosystems remains a challenge.

Heavy metals (HMs) are “trace elements” in pristine ecosystems, whereas their concentrations increase when there are anthropogenic activities (steel industry, waste disposal, agriculture, mining). HMs pose a risk owing to their toxicity, bioaccumulation and biomagnification (Alloway, 2013); they cause cell oxidative stress through formation of free radicals and are able to replace essential metals in pigments or enzymes, with consequent disruption of their functions (Ancona et al., 2020). PCBs and HMs are frequently found as soil co-contaminants (Panagos et al., 2013; Ancona et al., 2017a), posing a risk for both ecosystem and human health. Widespread and chronic environmental contamination by PCBs and HMs requires remediation solutions in line with environmental sustainability. The European Commission recently proposed a new priority and environmental friendly approach termed “Nature-Based Solutions, NbS” (European Commission, 2016; Faivre et al., 2017), relying on increasing and exploiting ecosystem homeostatic natural capacities to recover from environmental impacts and providing at the same time social and economic benefits. In this context, natural microbial communities have, thanks to their wide metabolic versatility and adaptation capacity, a key role in ecosystem regulation services, ensuring geochemical cycles and removing chemicals from contaminated environments (Turbé et al., 2010). The presence of an abundant and diverse microbial community with the ability to remove contaminants is a necessary prerequisite for an immediate and effective response to the various contaminants continuously affecting ecosystems (Barra Caracciolo et al., 2013). The changes that can be observed in a microbial community can be analyzed from a global and multivariate perspective, to understand and assess the impact of a complex chemical mixture on both sensitive microbial populations and resistant and/or resilient ones (Saccà et al., 2019). Although the metabolic potential of microbial communities is unlimited, some recalcitrant compounds such as PCBs require various abiotic conditions (e.g., both aerobic and anaerobic conditions, and electron donors) for biodegradation, and these are difficult to achieve in one and the same environment (Di Lenola et al., 2018). To improve natural biodegradation of chemicals, Plant-Assisted Bioremediation (PABR) has been proposed in recent years for the restoration of contaminated soils (Licht and Isebrands, 2005; Zalesny et al., 2016; Mercado-Blanco et al., 2018). PABR is an *in situ* treatment where plants are used for stimulating the bacterial degradation of persistent organic contaminants such as polychlorinated biphenyls (PCBs) and phyto-stabilization of inorganic ones (e.g., HMs) (Gamalero et al., 2012; Ancona et al., 2017a). The complex and synergistic actions established in the rhizosphere between the tree root system and natural belowground microbiota make it possible to remove, convert or contain toxic substances in soils (Aken et al., 2010; Ancona et al., 2020). Our previous samplings in a contaminated area we studied showed the ability of the hybrid poplar genotype Monviso (*Populus generosa* × *Populus nigra*) to grow in a nutrient poor soil, resisting high metal concentrations and promoting PCB biodegradation in the rhizosphere. The Monviso clone was effective in promoting both an overall contaminant decrease (PCB degradation and HM phyto-stabilization) and an increase in soil microbial activity, a little more than 1 year after planting the trees (Ancona et al., 2017a, b). The bioremediation strategy has continued until now and the results of subsequent chemical (PCBs and HMs) and microbiological analyses at 55 months are reported in the present study. In particular, the microbial community was analyzed in detail by determining the total microbial abundance, the cell viability and the composition of the bacterial groups using Next Generation Sequencing (NGS). The environmental DNA and RNA were used as templates for sequencing the hypervariable V4–V5 regions of 16S rRNA using MiSeq Illumina. The microbiological analyses were performed in the planted plots (both Rhizosphere and Bulk Soil samples) and un-planted soil (Control samples). The shifts in the structure and function (functional profiling predicted by PICRUSt2) of the belowground microbial community in the planted plots (poplar Rhizosphere and Bulk Soil immediately close to it) are reported.

## Materials and Methods

### Study Area and Sampling

The area (≈4.5 Ha) near the city of Taranto in Puglia (Italy) had been contaminated for several decades by wastes and pollutants (mainly HMs and polychlorinated biphenyls) because it was used as an illegal city dump. In particular polychlorinated biphenyls (PCBs) accumulated over time due to spilling and improper disposal of dielectric fluids from a power station with transformers nearby. A preliminary characterization of the experimental site showed PCB hot spots and widespread HM contamination with values higher than the legal limits, as reported in detail in Ancona et al. (2017a). In April 2013 about 650 poplar cuttings were planted in a site sub-area of 785 m^2^, in 8 rows 2 m apart. Inside each row the cuttings were placed at a distance of 0.5 m from each other. A first sampling campaign performed at 14 months from their planting showed a general decrease in PCBs and HMs thanks to the poplar-assisted bioremediation strategy applied (Ancona et al., 2017a). In the present study we report the results of a further sampling performed at month 55 after planting. As in the previous sampling, three contaminated plots were sampled. They were selected for the highest initial values of PCB contamination (i.e., average values of about 240 ng/g), which exceeded the national legislative limit (Italian Decree152/06) of 60 ng/g for green areas (Ancona et al., 2017a).

The area of each plot was 1 m^2^, with the plant at its center. In each plot, soil sampling was performed in two points: Rhizosphere and Bulk Soil. Aliquots (500 g) of the soil immediately surrounding the roots and in correspondence of the tree (5–30 cm depth) were collected with sterilized scoops and spatulas to have “Rhizosphere” samples. In the case of the Bulk Soil (50 cm distance from the trunk and at 20–40 cm depth), four soil aliquots (500 g) were sampled, by using a diamond coring machine (DD 130-W Core drill, HILTI, Germany) and mixed to obtain a composite soil. Moreover, four soil (500 g) samples were collected from an un-planted plot outside the plantation (ca 20 m distant), blended and used as Control. The details of the sampling procedure is reported in Ancona et al., 2017a.

From each plot, two composite sub-samples (2 g each) were immediately stored in a Life-Guard Soil Preservation Solution (Qiagen, Manchester, United Kingdom) in order to halt any possible degradation of the microbial RNA and consequently preserve the gene expression profiles and microbial community structure composition of any active cells. In a similar way, other two soil sub-samples (1 g each) were immediately fixed in a phosphate buffer saline solution with formaldehyde 2% for DAPI counts. Finally, two fresh soil sub-samples (1 g each) were used for evaluating the microbial activity.

Two composite samples (1,000 g each) were also collected for chemical analysis and stored at room condition. Each datum from each different condition (Rhizosphere, Bulk Soil and Control) is reported as an average value of the three replicate plots.

### PCB Chemical Analysis

Polychlorinated biphenyls were extracted from soil samples (Rhizosphere, Bulk and Control) with an Accelerated Solvent Extractor (ASE 300 DIONEX, United States). About 500 mg of powdered soil were mixed with previously washed diatomaceous earth to form a free-flowing powder. The mixture was extracted in an ASE extraction cell and purified using Silica Gel activated with concentrated sulfuric acid (3:2) in accordance with 210 DIONEX Technical note. The extraction solvent used was n-hexane operating at 100°C and 1,500 psi. The organic extracts were then evaporated to incipient dryness under a gentle nitrogen stream (using a Caliper Life Sciences TurboVap II Concentration Workstation) and re-solubilized into 0.5 mL Nonane. GC-MS analysis of 31 PCB congeners (Supplementary Table S5, Supplementary Material 5) was performed using a Thermo Electron TRACE GC Ultra coupled with a PolarisQ Ion Trap (Thermo Electron, Austin, TX, United States) mass spectrometer equipped with a PTV injector and a TriPLUS RSH autosampler. Details for GC-MS analyses are described in Supplementary Material 5. PCB quantification analysis showed that only 8 congeners – 6 PCB markers and two dioxin-like ones (105, 118) – were detected in the soil samples collected at different times of investigation (*t* = 0 and *t* = 55 months).

### Heavy Metals Analysis

Soil samples were acid digested in closed PTFE vessel devices using temperature control microwave heating (Ethos Touch Control, Milestone, Microwave Laboratory Systems). Soil mineralization was performed using Aqua Regia extraction by treating 500 mg of powdered samples with 9 mL of concentrated HCl and 3 mL of HNO_3_ (Madejón et al., 2004). Heating was achieved in a two-step procedure: 10 min to reach 200°C followed by 15 min at 200°C. Resulting solutions were diluted with ultrapure water in order to obtain a maximum content of 5% of acids and of 0.2% dissolved solids.

The quantification of 14 different mineral elements (Be, V, Cr, Co, Ni, Cu, Zn, As, Se, Cd, Sn, Sb, Tl, Pb) was performed by mass spectrometry with an inductively coupled plasma source (ICP-MS) equipped with a 7700× Agilent (Agilent Technologies, Japan).

### Organic Carbon and Total Nitrogen Analysis

Total and organic carbon and total nitrogen were determined with an elemental analyser (Carlo Erba NA 1500 series 2 C/H/N/O/S) equipped with an autosampler. For the organic carbon analyses, the samples were acidified with 20 μ l5 M ultrapure HCl and kept at 50–60°C for 30 min in order to remove inorganic carbon. The method used is based on the complete and instantaneous oxidation of the solid sample by combustion in an O_2_ enriched environment. Each sample, contained in tin or silver capsules for the determination of total and organic carbon, respectively, was introduced into the combustion reactor and maintained at about 1000°C for 20 min. Helium was used as a carrier gas to transport the combustion gases (CO_2_, N_2_, H_2_O) through a catalyst (Al_2_O_3_-WO_3_) in order to inhibit the formation of nitrogen.

The combustion mixture, together with the excess of oxygen, passed through a reduction column, consisting of granules of pure reduced copper, maintained at about 650°C, where the excess oxygen was adsorbed and any nitrogen oxides present were transformed into elemental nitrogen. The resulting gases (CO_2_, N_2_, H_2_O) then passed through an absorbent filter (magnesium perchlorate), which retains the water, to a gas chromatographic column that separates N_2_ from CO_2_. The column connected to a thermal conductivity detector (TCD) provided an output signal proportional to the quantity of the individual components in the mixture. From the measurement of the chromatographic peak area, corrected for white, the amount of carbon and nitrogen was obtained by comparison with a calibration curve.

### Total Microbial Abundance and Cell Viability

Microbial abundance and cell viability were analyzed using epifluorescence microscope-based methods which do not need DNA extraction from soil. The microbial abundance (No. cells/g dry soil) was assessed performing total direct counts. This method makes it possible to detect all the microbial cells present in a soil sample regardless of their physiological state and metabolic activity, thanks to the DAPI dye (4′,6-diamidino-2-phenylindole), which is a DNA fluorescent intercalant. Fixed soil subsamples (1 g each) were processed as reported in detail in Barra Caracciolo et al. (2005).

The cell viability (% live cells/live + dead) was evaluated in fresh soil subsamples (1 g each). The two dyes used to measure the ratio of live to dead cells were propidium iodide and SYBR Green II (Sigma-Aldrich, Germany) respectively, as described in detail in previous works (Grenni et al., 2009, 2012).

### DNA and RNA Extraction and Sequencing

Extraction of environmental RNA and DNA (eDNA) from each soil sample (Rhizo, Bulk and Control) was carried out using the RNA Power Soil Total RNA Isolation Kit and the DNA Elution Accessory kit (Qiagen), following the manufacturer’s recommendations. In order to evaluate the putative active bacteria in Rhizosphere and Bulk Soil, the same soil samples were used for RNA extraction; the latter was extracted as reported above and ds-cDNA synthesized in accordance with (Lasa et al., 2019b). Both eDNA and ds-cDNA were used as templates for MiSeq Illumina sequencing of the hypervariable V4–V5 region of the 16S rRNA gene, using the U519F and U926R primers (Baker et al., 2003).

The amplification with MiSeq Illumina was performed by Bio-Fab Research s.r.l. (Rome, Italy).

### Data Processing

A total of 10 Mb reads were generated and were analyzed using SEED2, a user interface-based sequence editor and NGS data analysis pipeline (Větrovský and Baldrian, 2013), and Mothur (Schloss et al., 2009) software packages. The forward and reverse reads were joined using SEED2 software with a 15% of maximal difference and minimal overlap of 40 bps. The primers, sequences with an ambiguous base and average quality score lower than 30 were removed. Before performing the chimera cleaning, with USEARCH^1^ from the SEED2 software package, we de-replicated the sequences. After the chimera cleaning the sequences were re-replicated. To avoid bias, the number of sequences in each sample was rarefied to the lowest one.

The resulting filtered and trimmed sequences were clustered into Operational Taxonomy Units (OTUs) using UPARSE (Edgar, 2013) from the SEED2 package and applying the average neighbor algorithm with a similarity cut off of 97%. The OTUs with less than 0.005% of high-quality reads were then filtered to avoid an overestimation of the diversity (Bokulich et al., 2013; Fernández-González et al., 2020).

With the Mothur software, the centroid sequence of each cluster was selected as the most representative of each OTU and was taxonomically classified at the genus level according to Ribosomal Database Project (RDP)^2^ trainset 16 with a confidence threshold of 80%.

The number of raw and filtered sequences is reported in SupplementaryTable S3.

### Predictive Functional Analysis

The PICRUSt2 software tool^3^ was used for predicting functional abundances based on the 16S rRNA gene amplicon data sets (Douglas et al., 2019); the OTUs table generated by UPARSE was used as an/the input. The prediction of KO relative abundances was performed with hidden-state prediction (Louca and Doebeli, 2018) and was used to infer pathways abundances (Ye and Doak, 2009). The statistical analysis was carried out with STAMP (version 2.1.3)^4^ in order to evaluate the significant differences between metagenome metabolic profiles under the different conditions.

### Statistical Analysis

An Alpha diversity table was created using SEED2 software with several diversity indices (Species richness, Shannon and Evenness indices). Principal Coordinate Analysis (PCoA) was performed with the online tool MicrobiomeAnalyst^5^ using a Bray-Curtis index dissimilarity method and PERMANOVA as the statistical method in order to analyze the Beta diversity. The *t*-tests were performed to determine if there was a significant difference between the means of two groups (between Rhizosphere and Bulk Soil and DNA and cDNA). All the histograms and the PCoA graphs were made using MS Excel.

## Results

### Effects of Plants on Pollutants Removal

**FIGURE 1:**
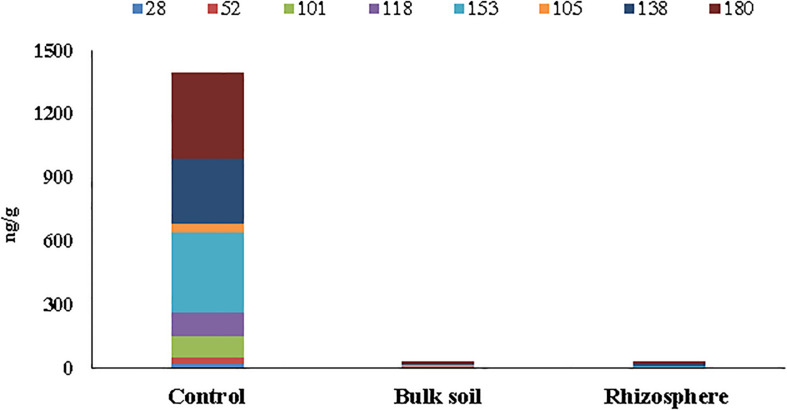
Concentrations (ng/g soil) of six PCB markers (28, 52, 101, 153, 138, and 180) and two dioxin-like (105 and 118) detected in soil samples collected at 55 months after poplar plantation.

The concentrations of six PCB markers (28, 52, 101, 153, 138, and 180) and two dioxin-like congeners (105, 118) are reported in Supplementary Table S1 (Supplementary Material 1) for unplanted (Control) and planted plots before the poplar plantation (*t* = 0) and at 55 months. In particular, for the planted plots, the analyses were performed in the Bulk Soil and in the Rhizosphere. Only residual concentrations of PCBs, below the national legal limit of 60 ng/g soil (Italian Decree 152/06), were found at 55 months in the planted plots (both in the Bulk Soil and the Rhizosphere samples). On the other hand, in the Control soil, outside the planted area, a high PCB concentration (∼1,400 ng/g soil) still persisted (Supplementary Table S1 Supplementary Material 1 and Figure 1).

**FIGURE 2:**
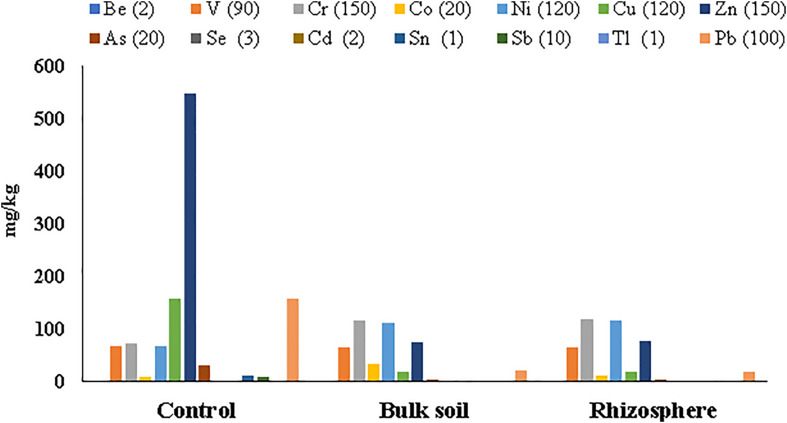
Heavy metal concentrations detected in soil samples collected at 55 months after poplar plantation. Italian law limits for each metal are in brackets.

Metal concentrations in soil samples from the planted plots (Rhizosphere and Bulk Soil) decreased if compared with the same soil before the planting (Control *t* = 0); all HMs were below the Italian legal limits, excepted for cobalt (Co) in the Bulk Soil (Supplementary Table S2, Supplementary Material 1). On the contrary, clear contamination still persisted in the un-planted soil (Control *t* = 55 months), where the highest metal concentration was found for Zn (549.73 ± 242.59 mg/kg) (Supplementary Table S2 Supplementary Material 1 and Figure 2).

### Microbiological Abundance, Organic Carbon, and Nitrogen Contents

**FIGURE 3:**
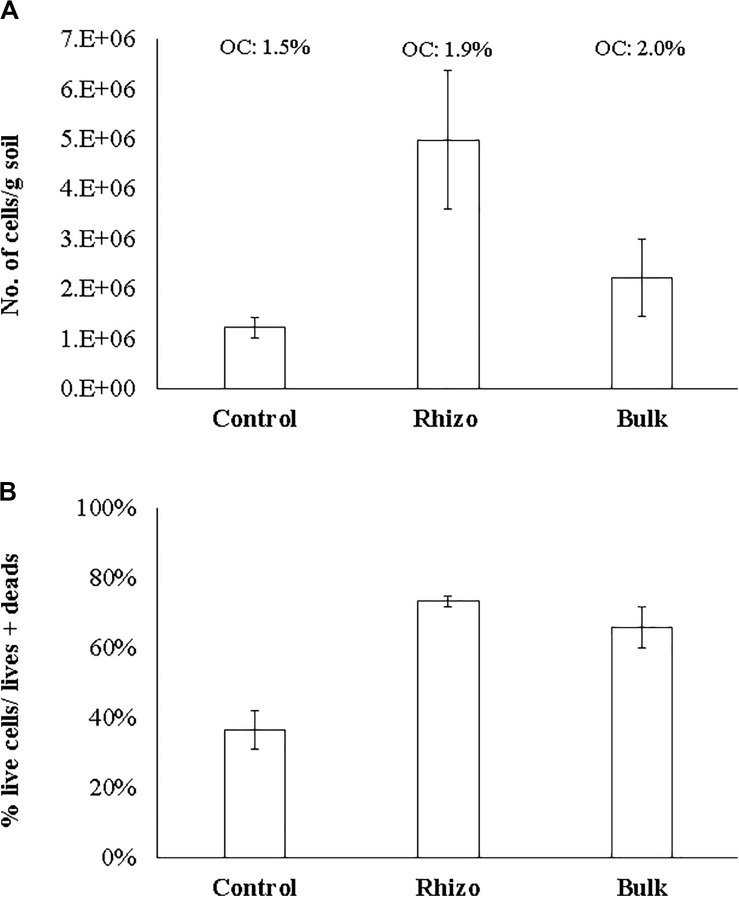
**(A)** Microbial abundance (No. cells/g soil); **(B)** Microbial viability (% live cells/live + dead) OC: organic carbon (%).

The microbial abundance (N. cells/g soil) and cell viability (% live cells/live + dead) were significantly (*t*-test, *p* < 0.01) higher in the planted plots (Rhizosphere and Bulk Soil) than in the Control. The highest values were found in the Rhizosphere (Figures 3A,B). Similarly, cell viability was higher (*t*-test, *p* < 0.01) in the Rhizosphere and Bulk Soil than in the Control (Figure 3B). In line with these results, the percentages of organic carbon were higher (*t*-test, *p* < 0.01) in the Rhizosphere (1.9% ± 0.11) and Bulk Soils (2.0% ± 0.10) than in the Control (1.5% ± 0.1).

Finally, the highest total nitrogen value (0.16% ± 0.001) was detected in the Rhizosphere, while lower values were found in the Bulk (0.12 ± 0.001) and Control soils (0.09 ± 0.001).

### DNA Sequencing Results

A total of 5,745,690 raw reads were obtained from the DNA (V4–V5 regions) deep-sequencing. After a trim and quality edit, 2,485,475 reads were retained: 21% of the total reads belonged to the Control, 41% to the Bulk Soil and 38% to the Rhizosphere. 99.9% of the total OTUs were classified at the Phylum level, 78.3% at the Class level, 66.6% at the Order level, 57.7% at the Family level and 39.0% at the Genus level, respectively.

**FIGURE 4:**
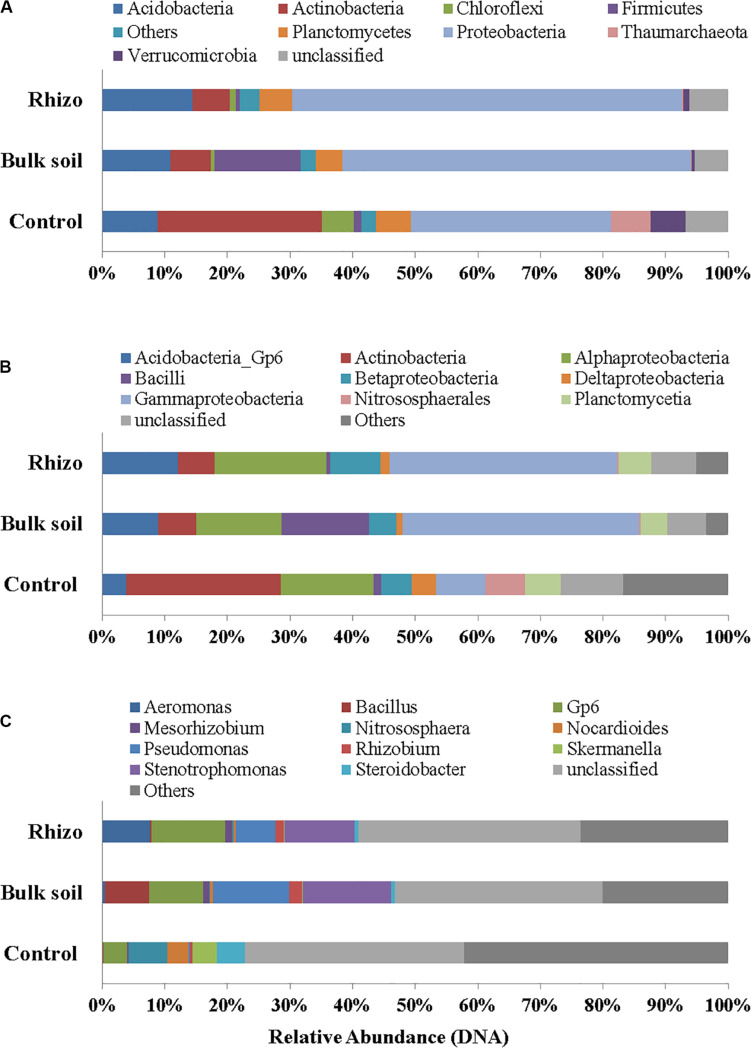
Prokaryotic communities in Rhizosphere (Rhizo), Bulk Soil (Bulk), and unplanted soil (Control). **(A)** Phylum level, **(B)** Class level, and **(C)** Genus level in DNA analysis.

*Proteobacteria* were the dominant Phylum both in the planted plots (62% in the Rhizosphere and 55% in Bulk Soil, respectively) and in the Control (32%); the latter percentage was significantly (*t*-test, *p* < 0.01) lower if compared to the Rhizosphere and Bulk Soil. *Acidobacteria*, *Firmicutes*, and *Actinobacteria* were the second most abundant group in the Rhizosphere (14.39%), Bulk Soil (13.70%), and Control (26%), respectively (Figure 4A). *Chloroflexi* (5%) and *Thaumarchaeota* (*Archaea*) (6%) were also present in the Control.

At the class level, the *Gammaproteobacteria* group was the most abundant one, both in the Rhizosphere and Bulk Soil (36 and 38%, respectively), while in the Control its percentage was significantly lower (8%), (*p* < 0.001). *Alphaproteobacteria* were present with a percentage of 18% in the Rhizosphere, and 13.5 and 15%, respectively in the Bulk and Control soils. *Betaproteobacteria* were 8% in Rhizosphere, and 4 and 5%, respectively in the Bulk and Control soils. *Acidobacteria* were 12% in the Rhizosphere, 9% in the Bulk soil and significantly lower in the Control (4%, *p* < 0.05). Finally, the percentage of *Actinobacteria* was higher in the Control (26%) than in the other conditions (6% in both Rhizosphere and Bulk Soil; *t*-test, *p* < 0.0001) (Figure 4B).

Figure 4C reports the most abundant genera found. A higher number of genera, most of them belonging to *Proteobacteria* (*Gammaproteobacteria*: *Aeromonas*, *Pseudomonas*, *Stenotrophomonas*, *Steroidobacter*; *Alphaproteobacteria*: *Mesorhizobium*, *Rhizobium*), were identified in the planted plots (Rhizosphere and Bulk Soil); higher percentages of Gp6 (*Acidobacteria*) and of *Nitrososphaera* (*Nitrososphaerales* – *Archaea*) were also found.

Some genera, such as *Nocardiodes* (*Actinobacteria*), *Skermanella* (*Alphaproteobacteria*), and *Steroidobacter* (*Gammaproteobacteria*), were found in higher percentages in the Control than in the planted plots. Finally, several genera were found in percentages lower than 1%. Interestingly, significant differences were found between the Control and planted soils, not least for the less abundant genera.

The community diversity of the planted plots (Rhizosphere and Bulk Soil) and Control soil were evaluated considering both the alpha (Table 1 for DNA and Table 2 for cDNA) and beta diversity (Supplementary Figures S1A,B, Supplementary Material 1).

**TABLE 1:** Average values of reads obtained from DNA and values of Alpha diversity expressed as Chao1, Shannon and Evenness indices in planted plots (Rhizosphere and Bulk Soil) and Control; ± standard errors.

DNA	Reads Filtered	Chao1	Shannon Index	Evenness
Rhizosphere	154,5645 ± 16,974	2,180 ± 183.79	9.68 ± 0.22	0.83 ± 0.01
Bulk Soil	173,528 ± 12,710	2,063 ± 102.70	9.10 ± 0.16	0.88 ± 0.01
Control	129,109 ± 6,442	2,855 ± 88.65	10.70 ± 0.08	0.93 ± 0.00

**TABLE 2:** Average cDNA read values and Alpha diversity values expressed as Chao1 Shannon and Evenness indices in planted plots (Rhizosphere and Bulk Soil); ± standard errors.

cDNA	Filtered Reads	Chao1	Shannon Index	Evenness
Rhizosphere	149,644 ± 15,393	2,045 ± 198.33	9.31 ± 0.19	0.85 ± 0.01
Bulk Soil	150,652 ± 15,641	1,334 ± 250.89	6.94 ± 0.77	0.67 ± 0.06

The Kruskal–Wallis test for the Chao1 index (Supplementary Table S4, Supplementary Material 1) showed significant differences between Control and Bulk Soil communities (*p* < 0.05); no differences were found between Rhizosphere and Bulk Soil and Rhizosphere and Control. The test performed for the Shannon Index found significant differences between Control and planted plots (Rhizosphere and Bulk Soil), (*p* < 0.02 both the samples). Finally, the Evenness index showed Control with a more homogenous community than Rhizosphere and Bulk Soil (*p* < 0.02).

The Bray–Curtis PCoA confirmed significant differences in the community structure under the different conditions: Control was significantly dissimilar from both Bulk Soil and Rhizosphere (PERMANOVA, *p* < 0.001). Moreover, a significant dissimilarity (PERMANOVA, *p* < 0.002) was also found inside each plot between the Rhizosphere and Bulk Soil samples (Supplementary Figure S1A, Supplementary Material 1).

### cDNA Sequencing Results

The microbial community was analyzed through amplicon sequencing of the 16S rRNA gene and the 16S rRNA transcripts to directly compare the total and active microbial communities (Roesch et al., 2007; De Vrieze et al., 2018; Lasa et al., 2019a) and to better identify the microorganisms potentially involved in the soil decontamination processes. In total 3,995,841 raw reads were obtained from cDNA sequencing and after a trim and quality edit, 1,801,772 were retained: 48% of them derived from the Bulk Soil and 52% the Rhizosphere, respectively. The average trimmed read values for each Rhizosphere and Bulk Soil are reported in Table 2.

The Kruskal–Wallis test on the alpha diversities indices showed that there were no significant differences for the Chao1 index, whereas species diversity and evenness were significantly higher in the Rhizosphere than Bulk Soil (respectively *p* < 0.02 and *p* < 0.01, Supplementary Table S4).

The PCoA analysis confirmed significant differences between the overall OTU data from DNA and cDNA and between the Rhizosphere and Bulk Soil cDNA (PERMANOVA, *p* < 0.001 Supplementary Figure S1B, Supplementary Material 1).

**FIGURE 5:**
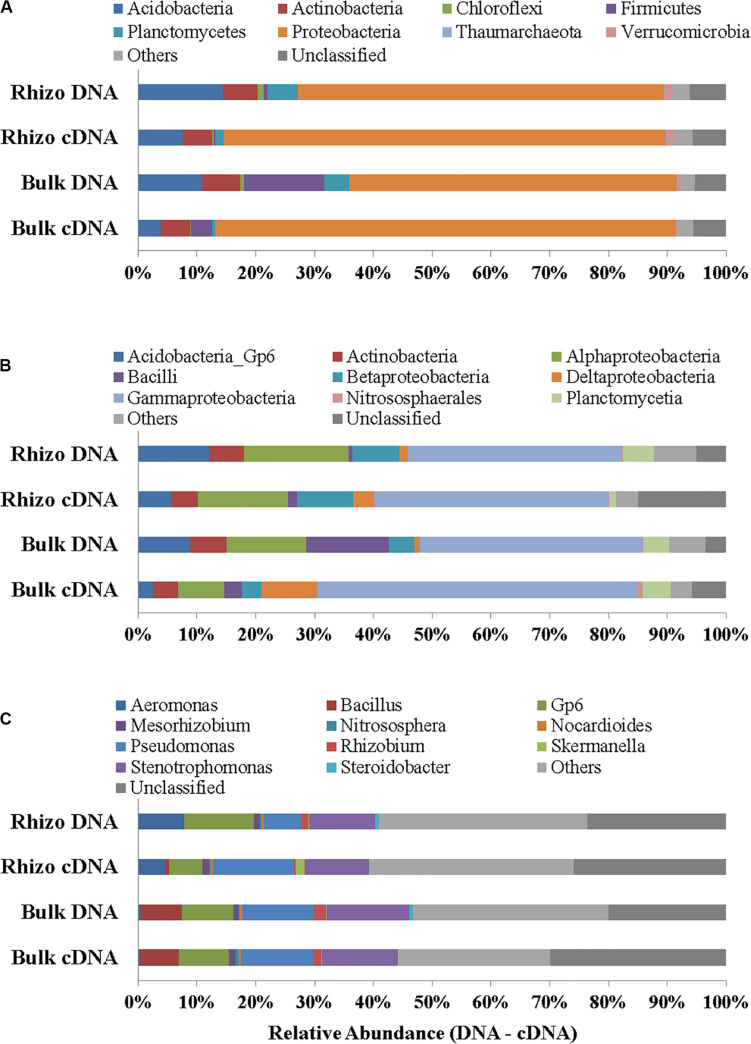
Prokaryotic communities in both DNA and cDNA in Rhizosphere (Rhizo) and Bulk Soil. **(A)** Phylum level, **(B)** Class level, and **(C)** Genus level.

In cDNA the dominance of *Proteobacteria* was even more evident (75% of the overall Prokaryotic community in Rhizosphere and 78% in Bulk Soil) than in DNA and quite low percentages of other phyla (such as *Acidobacteria*, *Actinobacteria*, *Firmicutes* and *Planctomycetes*) were found, in both the Rhizosphere and Bulk Soil samples (Figures 5A–C). The genera identified in the Rhizosphere (119) and in the Bulk Soil (118) are reported in Supplementary Material 2. Several genera were present in different percentages when comparing the Rhizosphere and Bulk Soil (e.g., *Pseudomonas* 14% Rhizosphere and 12% Bulk Soil, *Stenotrophomonas* 11% Rhizosphere and 13% Bulk Soil, *GP6* 6% Rhizosphere and 8% Bulk Soil and *Aeromonas* 5% Rhizosphere and less than 1% Bulk Soil).

### Functional Profiling of the Microbial Community

The functions of the bacterial communities in the samples were predicted by PICRUSt2, based on KEGG pathways using both DNA and cDNA sequences.

A total of 7,718 KEGG Orthologs (KOs) and 440 pathways were identified (Supplementary Material 3 and Supplementary Material 4). The PCA of the predicted functional profiling of the soil microbial community based on the DNA samples showed notable differences in the basic cellular processes between the various conditions (Rhizosphere, Bulk Soil and Control, Supplementary Figure S2A, Supplementary Material 1). This result demonstrated that the poplars influenced not only the structure (i.e., bacterial diversity), but also the functioning of the soil bacterial community.

**FIGURE 6:**
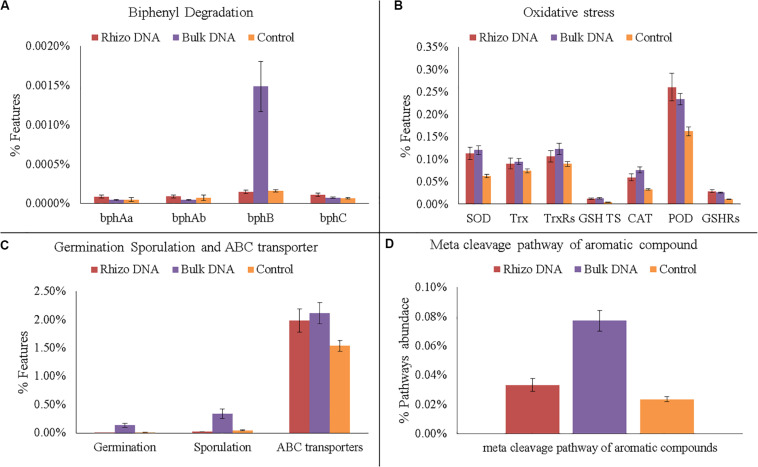
Functional gene analyses on DNA data. **(A)** Number of gene families encoding enzymes of the biphenyl degradation: biphenyl 2,3-dioxygenase (*bphAa*, *bphAb*); *cis*-2,3-dihydro-2,3-dihydroxybiphenyl dehydrogenase (*bphB*); 2,3-dihydroxybiphenyl 1,2-dioxygenase (*bphC*). **(B)** Number of gene families encoding enzymes and proteins related to oxidative stress: superoxide dismutase (SOD); thioredoxin (Trx); thioredoxin reductase (TrxRs); glutathione transport system (GSH TS); Catalase (CAT); peroxidase (POD); glutathione reductase (GSHRs). **(C)** Number of genes families related to germination, sporulation and ABC transporters. **(D)** Abundance of meta cleavage pathway of aromatic compounds.

**FIGURE 7:**
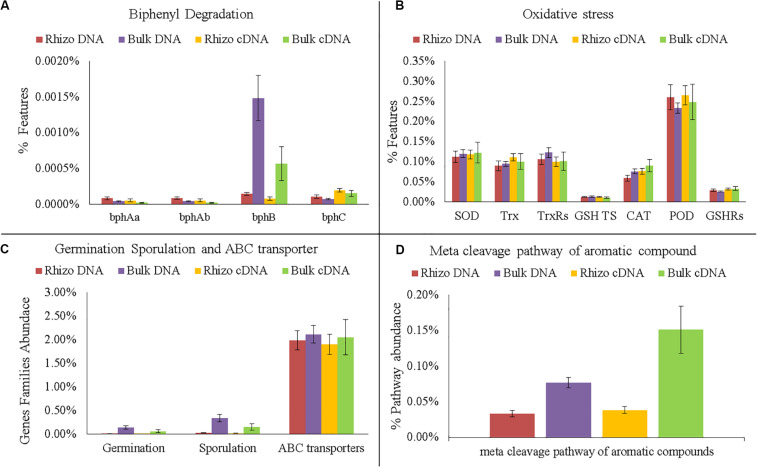
Comparison of functional gene analyses on DNA and cDNA samples. **(A)** Number of gene families encode enzymes of the biphenyl degradation: biphenyl 2,3-dioxygenase (*bphAa*, *bphAb*); *cis*-2,3-dihydro-2,3-dihydroxybiphenyl dehydrogenase (*bphB*); 2,3-dihydroxybiphenyl 1,2-dioxygenase (*bphC*). **(B)** Number of gene families encoding enzymes and proteins related to oxidative stress: superoxide dismutase (SOD); thioredoxin (Trx); thioredoxin reductase (TrxRs); glutathione transport system (GSH TS); Catalase (CAT); peroxidase (POD); glutathione reductase (GSHRs). **(C)** Number of Gene families related to germination, sporulation and ABC transporters. **(D)** Abundance of meta cleavage pathway of aromatic compounds.

In any case, various genes related to PCB degradation, HM and oxidative stress, membrane transporters, germination and sporulation were identified in both the planted plots and Controls (Figures 6A–D, 7A–D). For example, four gene families encoding enzymes involved in biphenyl degradation were found: *bphAa*, *bphAb* (biphenyl 2,3-dioxygenase), *bphB* (*cis*-2,3-dihydro-2,3-dihydroxybiphenyl dehydrogenase), and *bphC* (2,3-dihydroxybiphenyl 1,2-dioxygenase) (K08689, K08690, K015750, K018087, respectively). However, *bphB* was found with the highest values in the planted plots and particularly in the Bulk Soil. Moreover, genes potentially involved in aerobic PCB degradation with the meta cleavage pathway of aromatic compounds (i.e., degradation to pyruvate and acetyl-CoA), were also identified and were most abundant in the planted plots (Bulk Soil and Rhizosphere).

As regards HMs, several genes associated with their presence were also identified. In fact, functional genes associated with the oxidative stress caused by HMs, such as catalase (CAT) superoxide reductase (SOR), peroxidase, glutathione reductase (GR), and thioredoxin (Trx), were identified with a higher relative abundance in the planted plots than in the Controls. Similarly, the highest relative abundance of germination and sporulation genes was found in the Bulk Soil. Finally, ABC transporters, which make cellular HM uptake and expulsion possible, were higher in the planted plots than in the Controls.

Interestingly, the PCA performed on cDNA (Supplementary Figure S2B, Supplementary Material 1) showed that the active microorganisms inside the planted plots were metabolically similar. Significant differences between the Rhizosphere and Bulk Soil were still found in the meta cleavage pathways of aromatic compounds and in *bphB*, which were more abundant in the Bulk Soil than in Rhizosphere (*t*-test *p* < 0.05) (Figure 7A).

## Discussion

Due to their low bioavailability and complex pathways, the degradation of PCBs in the soil is normally limited (Xu et al., 2010). For this reason, a poplar-assisted bioremediation strategy was applied in this contaminated area. This paper, to our knowledge, is one of the first to characterize the belowground microbial community in a soil decontaminated through PABR. The data showed an overall improvement in soil quality not only in terms of decontamination and increase in nutrients, but also in the structure and functions of the microbial community. The comparison of the chemical (PCBs and HMs) and microbiological analyses between control and planted plots showed the effectiveness of the PABR in recovering the contaminated area, considering both the soil immediately surrounding the roots (Rhizosphere) and the Bulk Soil at 50 cm from the trunk and at 40 cm depth. These results confirm the decontamination found at 14 months, although in the previous sampling some HMs (i.e., V, Sn and Pb) still exceeded their legal threshold values (Ancona et al., 2017a). At 55 months, the roots grown in the planted plots increased the volume of the rhizosphere habitat and positively influenced the entire belowground microbial community. In fact, at 55 months the poplar trees reached 6 m in height and the belowground root biomass had been able to spread fourfold compared to the previous 14-month sampling, reaching a volume of c.a. 16,000 cm^3^. This means that the planting layout of the poplars (0.5 × 2 m) was effective in ensuring that their root development positively stimulated the overall microbial community in each planted plot. The positive “rhizosphere effect” has been reported by several authors (Thomas and Cébron, 2016; Lasa et al., 2019b; Schneijderberg et al., 2020) and was presumably due to the root surface releasing several organic compounds into the adjacent soil. Roots release low-molecular-weight exudates (e.g., sugars, polysaccharides, amino acids, aromatic acids, aliphatic acids, fatty acids, sterols, phenolic compounds, enzymes, proteins, plant growth regulators, and secondary metabolites) which attract soil microorganisms into the rhizosphere, where they multiply by several orders of magnitude (Badri et al., 2009). Root microbiome comprises thousands of taxa among which are commensal, pathogens and beneficial organism. Plants can establish complex interactions in the rhizosphere and inside the root (endorhizosphere) with beneficial microbes (Schneijderberg et al., 2020). In turn, microorganisms strongly influence plant development and health by mineralizing organic matter, degrading organic pollutants, including persistent ones such as PCBs, improving HM bioavailability by varying the degree of reaction of the soil (pH), releasing chelating substances (organic acids, siderophores) and promoting oxidation/reduction reactions (Ancona et al., 2017a, 2020). A hierarchy of events determines the composition of the belowground microbiome of a plant: the first determinant is the initial microbial composition and heterogeneity of the soil. Then, plant species properties, including morphology, developmental, rhizodeposition and plant genotype, which will determine the microbial species composing a root microbiome (Philippot et al., 2013; Sasse et al., 2018). In line with the above, a shift in the belowground microbial community structure was shown by the significant differences in diversity indices (e.g., Shannon and Evenness) between Control and planted plots. Moreover, the comparison of the diversity inside planted plots showed significantly higher values in Rhizosphere than in Bulk Soil. This latter result suggests that the area immediately surrounding the roots can support a higher number of active cells.

Moreover, the DNA sequencing data showed higher percentages of bacteria genera (i.e., *Stenotrophomonas*, *Pseudomonas*, *Mesorhizobium*, *Rhizobium*, *Skermanella*, and *Gp6*), involved in biogeochemical cycles (in line with an increase in soil C and N) and xenobiotic degradation (Figure 5C), in the Rhizosphere and Bulk Soil than in the Control (Luo et al., 2008; Uhlik et al., 2012; Dudášová et al., 2014; Teng et al., 2016; Horváthová et al., 2018). On the other hand, the Control microbial community was quite different from that of the planted plots and some genera such as *Nitrososphaera* (*Archaea*), *Nocardioides* (*Actinobacteria*), and *Stereoidobacter* (*Gammaproteobacteria*) were significantly more abundant than in the other condtions.

The fact that the plant presence promoted a *Proteobacteria* dominance in all plots was demonstrated by both DNA (average value 58%) and even more by cDNA (avergae value 77%) results. The *Proteobacteria* increase was in line with the rise in microbial abundance and viability, showing their active role in removing chemicals and increasing soil quality. The comparison of DNA and cDNA showed that in fact different bacterial genera or percentages of each taxonomic group were active in the soil studied, highlighting not only the role of *Proteobacteria*, but also some differences in the percentages of some classes between the bulk soil and rhizosphere. The *Proteobacteria* group includes most bacterial species involved in the main biogeochemical cycles and is typically the most abundant phylum found in a good quality state soil (Barra Caracciolo et al., 2015) and several *Alpha*, *Beta*, and *Gammaproteobacteria* species have been reported to be able to degrade PCBs and increase with a plant presence (Praveckova et al., 2016; Di Lenola et al., 2018). Interestingly, in planted plots a higher percentage of Firmicutes than in Control was also found and in this group there are several bacteria able to resist and remove HMs (Pan et al., 2017; Fajardo et al., 2019; Johnson et al., 2019) and transform high-chlorinated PCBs anaerobically (Smidt and de Vos, 2004).

The DNA predictive functional analysis confirmed overall significant differences between the planted plots and Control (Supplementary Figure S2A, Supplementary Material 1). That it is to say that the poplars were able to change both the structure and functioning of the belowground microbial community. However, the predictive functional analysis showed that some genes involved in biphenyl degradation were present inside the control microbial community, demonstrating that the latter was potentially able to degrade PCBs and that the plant stimulated the specific microbial populations able to recover the soil. Interestingly, the cDNA predictive functional analysis showed that the overall bacterial populations inside each planted plot (Rhizosphere and Bulk Soil) were metabolically similar, showing how the plant positively influenced the functioning of the overall microbial community. In fact, the number of bacteria associated with some specific genes (e.g., *bphA*, *bphB*, and *bphC*) significantly increased in the planted plots (Rhizosphere and Bulk Soil), demonstrating the effectiveness of the poplar in promoting microbial transformations. *bphA* is a multi-component enzyme consisting of terminal dioxygenase and electron transfer components, which starts the degradation process of the biphenyl ring; *bphB* catalyzes the conversion of dihydrodiol to a dihydroxy compound and *bphC* is involved in ring meta-cleavage. Chlorobenzoate compounds are one of the final products of aerobic PCB degradation and can be degraded through the meta-cleavage pathway of aromatic compounds to pyruvate and acetyl-CoA (Chatterjee et al., 1981). In line with these results, numerous species of *Pseudomonas*, the dominant genus found in the planted plots, have these genes (Sala-trepat and Evans, 1971; Hughes and Bayly, 1983; Kukor and Olsen, 1991; Shingler et al., 1992; Xu et al., 2003). The fact that both *bphB* and the meta-cleavage pathway of aromatic compounds had higher values in the Bulk Soil than in the Rhizosphere shows that some degradation steps were presumably more favored in proximity of the rhizosphere.

The presence of HMs is known to cause bacterial physiological stress with the production of reactive oxygen species (ROS) (Choudhary et al., 2007). In this work, we found an increase in the planted plots in bacteria able to resist HM stress by producing these protective enzymes. In fact, many bacteria are able to reduce oxidative stress by producing CAT, superoxide reductase (SOD), peroxidase, glutathione reductase (GR) Trx, and thioredoxin reductase (TrxRs) (Banerjee et al., 2015). CAT and SOD are able to convert ROS into oxygen and water and make the maintaining of cellular integrity possible. SOD, a metalloenzyme which converts highly toxic superoxide into oxygen and less toxic hydrogen peroxide, is similar (Banerjee et al., 2015).

Similarly, we found an increase in the planted plots in bacteria able to respond functionally to toxic molecules using ABC transporters. Prokaryotic ABC transporters have a key role in the import of nutrients and export of unwanted molecules such as toxic ones (Fajardo et al., 2019). The overall functional profiling of the microbial community showed how the plant presence favored an increase in bacterial genera able at the same time to transform and degrade all contaminants and to respond functionally in order to maintain their homeostasis (Fajardo et al., 2019).

## Conclusion

This work has investigated the shifts in the natural microbial community in a soil where PCBs and HMs were removed by PABR. The overall results showed how the synergic relationships established between plants and belowground microorganisms were able to increase soil quality, by enriching the soil with active bacteria able to degrade and contain pollutants, resist stresses and increase soil nutrient content.

At the same time the poplar trees were able to grow healthily and transform a degraded area into one that could be used advantageously. Plants and their microbiota can be considered “metaorganisms,” i.e., able to overcome biotic and abiotic stress. The interactions of plants and belowground microbiota are complex and further studies are desirable for better investigating the overall process of contaminant removal, taking also in consideration the challenging study of endorizhosphere microbes.

The PABR strategy is recommended as an effective and good example of a nature-based solution, as mentioned and recommended by the European Commission.

## Data Availability Statement

The datasets presented in this study can be found in online repositories. The names of the repository/repositories and accession number(s) can be found in the article/ Supplementary Material.

## Author Contributions

All authors listed have made a substantial, direct and intellectual contribution to the work, and approved it for publication.

## Conflict of Interest

The authors declare that the research was conducted in the absence of any commercial or financial relationships that could be construed as a potential conflict of interest.

## Footnotes

1 http://www.drive5.com/usearch

2 http://rdp.cme.msu.edu

3 https://github.com/picrust/picrust2

4 https://pypi.org/project/STAMP

5 http://www.microbiomeanalyst.ca

## Abbreviations

CAT: catalase
GSH TS: glutathione transport system
GSHRs: glutathione reductase
HM: heavy metal
NGS: next-generation sequencing
POD: peroxidase
SOD: superoxide dismutase
Trx: thioredoxin
TrxRs: thioredoxin reductase.
