# Supplementary material for: Characterization of the Belowground Microbial Community in a Poplar-Phytoremediation Strategy of a Multi-Contaminated Soil
Source: Front Microbiol. 2020 Aug 25;11:2073. doi: 10.3389/fmicb.2020.02073 (PMC7477336; doi:10.3389/fmicb.2020.02073)
Supplement: Supplementary file 1 [file Data_Sheet_1.docx]

Supplementary Material1

**Table S1**. PCB congener concentrations (average values expressed as ng/g ± standard deviation) detected in the soil samples (Rhizosphere and Bulk Soil) collected in the investigated plots (planted and un-planted) before and 55 months after poplar plantation.

| **SOIL SAMPLES** | | **PCB markers (ng/g)** | | | | | | **PCB dioxin like (ng/g)** | | **TOTAL**  **(ng/g)** |
| --- | --- | --- | --- | --- | --- | --- | --- | --- | --- | --- |
|  |  | **28** | **52** | **101** | **153** | **138** | **180** | **105** | **118** |  |
| **UN-PLANTED PLOT** | **Control Soil 0 day** | 7.65±2.26 | 33.21±9.05 | 92.41±31.32 | 405.76±182.45 | 273.59±103.60 | 458.20±203.22 | 45.51±4.79 | 85.53±14.11 | 1,401.85±550.81 |
|  | **Control Soil 55 months** | 19.87±28.16 | 29.56±24.34 | 101.06±71.12 | 378.40±249.50 | 305.05±193.70 | 406.51±255.55 | 45.62±35.90 | 108.24±75.83 | 1,394.31±934.09 |
| **PLANTED PLOT** | **Bulk Soil**  **0 day** | 7.25±0.07 | 11.90±0.42 | 10.35±0.77 | 59.75±1.76 | 73.25±3.18 | 42.15±0.77 | 15.55±1.34 | 25.75±0.35 | 245.95±8.69 |
|  | **Rhizosphere 55 months** | 0.76±0.37 | 1.60±0.62 | 0.56±0.44 | 5.47±1.42 | 9.86±3.35 | 10.26±3.07 | 0.59±0.46 | 1.26±0.32 | 30.37±10.03 |
|  | **Bulk Soil**  **55 months** | 0.48±0.04 | 1.47±0.57 | 1.31±0.33 | 5.15±0.47 | 8.80±0.75 | 8.52±0.70 | 1.18±0.21 | 1.81±0.52 | 28.72±3.59 |

**Table S2**. Heavy metal concentrations (average values expressed as mg/kg ± standard deviation) detected in the soil samples (Rhizosphere and Bulk Soil) collected in the investigated plots (planted and un-planted) before and 55 months after poplar plantation. The Italian legal limits in mg/kg (D.Lgs. 152/06) are in brackets; the red values are those higher than the legal limit.

| **Sample** | | **Heavy metals (part A) expressed in mg/kg** | | | | | | | |
| --- | --- | --- | --- | --- | --- | --- | --- | --- | --- |
|  |  | **Be**  **(2)** | **Se**  **(3)** | **Cd**  **(2)** | **Tl**  **(1)** | **Sn**  **(1)** | **Sb**  **(10)** | **Co**  **(20)** | **As**  **(20)** |
| **UN-PLANTED PLOT** | **Control Soil 0 day** | 3.80±0.00 | **15.30**±3.50 | 1.50±0.00 | 0.80±0.00 | **24.80**±2.80 | 8.90±0.00 | 17.8±1.40 | **45.10**±5.00 |
|  | **Control Soil 55 months** | 0.97±0.09 | 1.40±0.11 | 0.91±0.32 | 0.37±0.05 | **12.83**±3.53 | 8.13±5.98 | 9.92±0.64 | **32.07**±14.47 |
| **PLANTED PLOT** | **Topsoil**  **0 day** | 1.90±0.20 | **10.40**±2.10 | 0.50±0.00 | 0.70±0.20 | **5.90**±2.10 | 4.70±0.10 | 19.40±2.10 | 17.60±3.20 |
|  | **Rhizosphere 55 months** | 0.92±0.04 | 1.37±0.15 | 0.18±0.01 | 0.26±0.01 | **1.77**±0.26 | 0.47±0.14 | 11.98±0.77 | 5.05±0.77 |
|  | **Bulk Soil**  **55 months** | 0.89±0.03 | 1.33±0.12 | 0.17±0.02 | 0.24±0.01 | **2.61**±1.15 | 0.54±0.10 | **34.26**±8.97 | 5.03±0.44 |

| **Sample** | | **Heavy metals (part B) expressed in mg/kg** | | | | | |
| --- | --- | --- | --- | --- | --- | --- | --- |
|  |  | **V**  **(90)** | **Ni**  **(120)** | **Cu**  **(120)** | **Cr**  **(150)** | **Zn**  **(150)** | **Pb**  **(100)** |
| **UN-PLANTED PLOT** | **Control Soil 0 day** | **123.8**±9.90 | 89.10±4.30 | **156.40±**11.40 | 132.70±15.60 | **767.30±**35.90 | **276.30**±26.90 |
|  | **Control Soil 55 months** | 67.16±9.45 | 68.33±3.44 | **158.93**±79.06 | 73.74±7.25 | **549.73**±193.70 | **159.30**±67.51 |
| **PLANTED PLOT** | **Topsoil**  **0 day** | **127.40±**17.90 | **156.20±**19.30 | 47.70±4.20 | **179.5±**20.70 | **181.30±**17.50 | 74.5±7.7 |
|  | **Rhizosphere 55 months** | 66.20±2.92 | 115.62±10.56 | 18.51±0.87 | 120.28±12.48 | 77.01±3.44 | 19.39±4.60 |
|  | **Bulk Soil**  **55 months** | 65.07±1.48 | 112.38±7.97 | 18.51±0.71 | 117.19±7.01 | 74.74±7.18 | 21.77±5.86 |

**Table S3.** Number of raw and filtered sequences for each sample and percentage (%) of retained sequences used for the analysis.

| **Sample** | **Sample ID** | **Type** | **Raw sequences input** | **Filtered sequences** | **Retained sequences (%)** |
| --- | --- | --- | --- | --- | --- |
| Bulk Soil | 5 | DNA | 410,785 | 207,854 | 51% |
| Bulk Soil | 6 | DNA | 370,848 | 183,510 | 49% |
| Bulk Soil | 7 | DNA | 379,682 | 198,703 | 52% |
| Bulk Soil | 8 | DNA | 261,449 | 121,023 | 46% |
| Bulk Soil | 11 | DNA | 316,610 | 160,904 | 51% |
| Bulk Soil | 12 | DNA | 315,309 | 169,176 | 54% |
| Control | E2B 17 | DNA | 491,170 | 147,757 | 30% |
| Control | E2B 18 | DNA | 426,358 | 118,788 | 28% |
| Control | S1Mons 19 | DNA | 470,779 | 136,304 | 29% |
| Control | S1Mons 20 | DNA | 448,483 | 113,584 | 25% |
| Rhizosphere | 9 | DNA | 299,101 | 157,327 | 53% |
| Rhizosphere | 10 | DNA | 264,109 | 134,169 | 51% |
| Rhizosphere | 13 | DNA | 277,640 | 124,137 | 45% |
| Rhizosphere | 14 | DNA | 467,711 | 235,730 | 50% |
| Rhizosphere | 15 | DNA | 291,419 | 147,611 | 51% |
| Rhizosphere | 16 | DNA | 254,237 | 128,898 | 51% |
| Bulk Soil | 21 | RNA | 301,780 | 159,266 | 53% |
| Bulk Soil | 22 | RNA | 243,092 | 103,250 | 42% |
| Bulk Soil | 23 | RNA | 323,470 | 151,724 | 47% |
| Bulk Soil | 24 | RNA | 238,323 | 119,069 | 50% |
| Bulk Soil | 27 | RNA | 310,475 | 156,848 | 51% |
| Bulk Soil | 28 | RNA | 508,191 | 213,753 | 42% |
| Rhizosphere | 25 | RNA | 314,268 | 145,060 | 46% |
| Rhizosphere | 26 | RNA | 431,658 | 187,687 | 43% |
| Rhizosphere | 29 | RNA | 427,891 | 192,226 | 45% |
| Rhizosphere | 30 | RNA | 252,958 | 97,042 | 38% |
| Rhizosphere | 31 | RNA | 342,161 | 157,748 | 46% |
| Rhizosphere | 32 | RNA | 301,574 | 118,099 | 39% |

**Table S4.** Kruskal-Wallis test paired for alpha indices (Chao1 index, Shannon Index, Evenness). The significant differences are in bold.

| **Chao1 Index** | | | | |
| --- | --- | --- | --- | --- |
| **Group 1** | **Group 2** | **H** | **p-value** | **q-value** |
| Control | Rhizosphere DNA | 3.682 | 0.055 | 0.128 |
|  | Bulk Soil DNA | 6.545 | **0.010** | 0.074 |
| Bulk Soil DNA | Rhizosphere DNA | 0.102 | 0.749 | 0.873 |
| Bulk Soil RNA | Rhizosphere RNA | 3.102 | 0.078 | 0.164 |
| **Shannon Index** | | | | |
| **Group 1** | **Group 2** | **H** | **p-value** | **q-value** |
| Control | Rhizosphere DNA | 6.545 | **0.010** | 0.037 |
|  | Bulk Soil DNA | 6.545 | **0.010** | 0.037 |
| Bulk Soil DNA | Rhizosphere DNA | 3.102 | 0.078 | 0.037 |
| Bulk Soil RNA | Rhizosphere RNA | 6.564 | **0.010** | 0.037 |
| **Evenness** | | | | |
| **Group 1** | **Group 2** | **H** | **p-value** | **q-value** |
| Control | Rhizosphere DNA | 6.545 | **0.010** | 0.037 |
|  | Bulk Soil DNA | 6.545 | **0.010** | 0.037 |
| Bulk Soil DNA | Rhizosphere DNA | 4.333 | **0.037** | 0.063 |
| Bulk Soil RNA | Rhizosphere RNA | 7.41 | **0.006** | 0.037 |


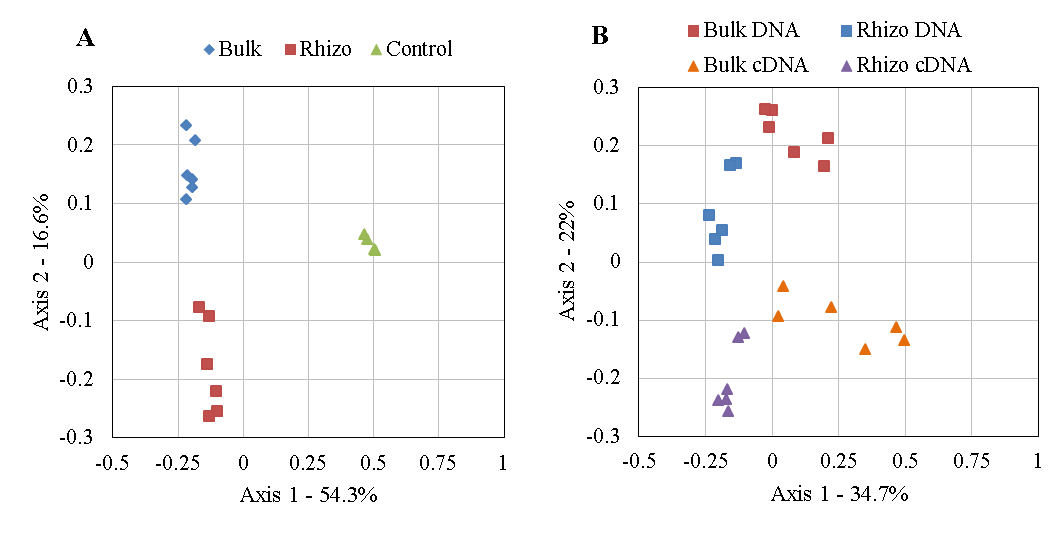


**FIGURE S1.** Bray-Curtis principal coordinate analisys at OTU level considering DNA (A, Permanova p < 0.001) and cDNA (B, Permanova p < 0.001) samples.


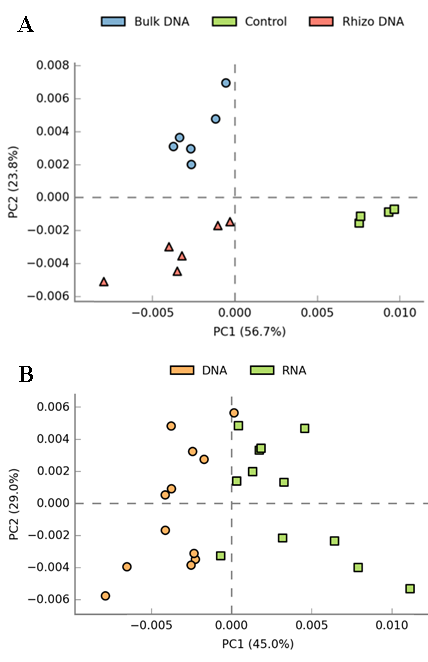


**FIGURE S2.** (A) PCA of functional genes profiling of DNA samples; (B) comparison of DNA and cDNA.
