# Supplementary material for: Characterization of the Belowground Microbial Community in a Poplar-Phytoremediation Strategy of a Multi-Contaminated Soil
Source: Front Microbiol. 2020 Aug 25;11:2073. doi: 10.3389/fmicb.2020.02073 (PMC7477336; doi:10.3389/fmicb.2020.02073)
Supplement: Supplementary file 5 [file Data_Sheet_5.docx]

Supplementary Material5

**PCB Analysis**

All solvents were of GC-MS grade: n-hexane (Carlo Erba ≥ 95%), nonane (≥ 99%), acetone (≥ 99.8 %). Diatomaceous earth sorbent (hydromatrix) was obtained from Dionex (Thermo Fisher Scientific, Sunnyvale, CA). All acids were of superpure grade for trace analysis: nitric acid (Carlo Erba 67-69 %), chloridric acid (Carlo Erba 34-37%) hydrogen peroxide (Carlo Erba 30%).

Mixtures of 31 PCBs (CB-18, CB-28, CB-52, CB-44, CB-95, CB-101, CB-99, CB-81, CB-77, CB-110, CB-151, CB-123, CB-149, CB-118, CB-114, CB-146, CB-153, CB-105, CB-138, CB-126, CB-187, CB-183, CB-128, CB-167, CB-177, CB-156, CB-157, CB-180, CB-169, CB-170, CB-189) congeners (C-WNN) were obtained from LabStandards as well as the internal standard [13C12]PCB 104). Standard Reference Materials of a mixtures of 14 elements (Be, V, Cr, Co, Ni, Cu, Zn, As, Se, Cd, Sn, Sb, Tl, Pb ) were obtained from Agilent (Multi-element calibration standards 2A) as well as the internal standard solution (Bi, Ge, In, Li, Lu, Rh, Sc and Tb).

**Instrumental Analysis**

*GC-MS*

GC-MS analysis of 32 PCBs (#18, 28, 44, 52, 77, 81, 95, 99, 101, 104*, 105, 110, 114, 118, 123, 126, 128, 138, 151, 146, 149, 153, 156, 157, 167, 169, 170, 177, 180, 183, 187, 189) including 6 PCBs indicators (#28, 52, 101, 138, 153, 180) and 12 dioxin-like (#77, 81, 126, 169, 105, 114, 118, 123, 156, 157, 167, 189) were performed using a ThermoElectron TRACE GC Ultra coupled with a PolarisQ Ion Trap (Thermo Electron, Austin, TX) mass spectrometer equipped with a PTV injector and a TriPLUS RSH auto-sampler. The system was managed by Thermo Electron Xcalibur software version 1.4.1. Compound separation was achieved using an Agilent CP8944 VF- 5ms U (length 30m, i.d. 0.25mm, film thickness 0.25µm) column.

All congeners were separated under the following conditions: carrier gas, helium with a constant flow rate of 1.4 mL/min; initial injector temperature, 75°C ramped to 280°C (held 2 min) at 14.5°C/s.

Two ions were monitored for each PCB homologous group using MS/MS acquisition mode (Table 1). The mass spectrometer was used in electronic impact (EI) mode (70 eV electron energy) with ion Source and transfer line temperatures of 260 and 250°C respectively. The oven temperature was initially set at 80°C for 2 min increased at first to 160°C at a rate of 25°C/min held for 1 min, then increased to 210°C at a rate of 4°C/min held for 10 min, further increased to 280°C at a rate of 10°C/min held for 1 min, and at last increased to 310°C at a rate of 30°C/min held for 1 min.

**Table S5**. Lists of PCB congeners and their Precursor and Product Ions used for MS/MS method for GC/MS analysis. *Compound is internal standard.

| **PCBs** | **Precursor Ion**  (m/z)  [Da] | **Product** **Ion**  (m/z)  [Da] |
| --- | --- | --- |
| CB-18 | 258 | 186, 188 |
| CB-28 | 258 | 186, 188 |
| CB-52 | 292 | 220, 222 |
| CB-44 | 292 | 220, 222 |
| CB-95 | 326 | 254, 256 |
| CB-101 | 326 | 254, 256 |
| CB-99 | 326 | 254, 256 |
| CB-81 | 292 | 220, 222 |
| CB-77 | 292 | 220, 222 |
| CB-110 | 326 | 254, 256 |
| CB-151 | 360 | 290, 288 |
| CB-123 | 326 | 254,256 |
| CB-149 | 360 | 290, 288 |
| CB-118 | 326 | 254,256 |
| CB-114 | 326 | 254,256 |
| CB-146 | 360 | 290, 288 |
| CB-153 | 360 | 290, 288 |
| CB-105 | 326 | 254,256 |
| CB-138 | 360 | 290, 288 |
| CB-126 | 326 | 254,256 |
| CB-187 | 395 | 324, 326 |
| CB-183 | 395 | 324, 326 |
| CB-128 | 360 | 290, 288 |
| CB-167 | 360 | 290, 288 |
| CB-177 | 395 | 324, 326 |
| CB-156 | 360 | 290, 288 |
| CB-157 | 360 | 290, 288 |
| CB-180 | 395 | 324, 326 |
| CB-169 | 360 | 290, 288 |
| CB-170 | 395 | 324, 326 |
| CB-189 | 395 | 324, 326 |
| CB-104* | 338 | 266, 268 |

*Quality control*

A known amount of labelled PCB standard (congener 104) was added to each sample prior to extraction and sometimes one OPR (Ongoing Performance Recovery) sample, consisting of a blank sample spiked with a known amount of labelled PCB standard and natives’ PCB, was also extracted and processed in order to check the performance of recovery of the GC-MS system.

PCBs recoveries were calculated for each sample. The samples analyzed were in the range of 55% to 105% recovery.
